# Relationship between 24-h venous blood glucose variation and mortality among patients with acute respiratory failure

**DOI:** 10.1038/s41598-021-87409-2

**Published:** 2021-04-08

**Authors:** Xiaoling Zhang, Jingjing Zhang, Jiamei Li, Ya Gao, Ruohan Li, Xuting Jin, Xiaochuang Wang, Ye Huang, Gang Wang

**Affiliations:** 1 Department of Critical Care Medicine, The Second Affiliated Hospital, Xi’an Jiaotong University, Xi’an, 710004 Shaanxi China; 2 Department of Emergency Medicine, Xi Yuan Hospital, China Academy of Chinese Medical Sciences, Beijing, 100091 China

**Keywords:** Risk factors, Outcomes research

## Abstract

Evidence indicates that glucose variation (GV) plays an important role in mortality of critically ill patients. We aimed to investigate the relationship between the coefficient of variation of 24-h venous blood glucose (24-hVBGCV) and mortality among patients with acute respiratory failure. The records of 1625 patients in the Multiparameter Intelligent Monitoring in Intensive Care II (MIMIC II) database were extracted. The 24-hVBGCV was calculated as the ratio of the standard deviation (SD) to the mean venous blood glucose level, expressed as a percentage. The outcomes included ICU mortality and in-hospital mortality. Participants were divided into three subgroups based on tertiles of 24-hVBGCV. Multivariable logistic regression models were used to evaluate the relationship between 24-hVBGCV and mortality. Sensitivity analyses were also performed in groups of patients with and without diabetes mellitus. Taking the lowest tertile as a reference, after adjustment for all the covariates, the highest tertile was significantly associated with ICU mortality [odds ratio (OR), 1.353; 95% confidence interval (CI), 1.018–1.797] and in-hospital mortality (OR, 1.319; 95% CI, 1.003–1.735), especially in the population without diabetes. The 24-hVBGCV may be associated with ICU and in-hospital mortality in patients with acute respiratory failure in the ICU, especially in those without diabetes.

## Introduction

Glucose variation (GV) has been identified as a risk factor for adverse outcomes in various patients^1^. A previous study indicated that increased standard deviation (SD) and coefficient of variation (CV), are independently associated with longer hospital stay and higher mortality in non-critically ill patients^2^. Besides, in critically ill patients, the higher mean absolute glucose (MAG) change over a given period or SD also conferred a strong independent risk of mortality^3–6^. However, the relationship between coefficient of variation of 24-h venous blood glucose (24-hVBGCV), calculated as the ratio of the SD to the mean of venous blood glucose readings available during first 24-h in ICU, and mortality among patients with acute respiratory failure in intensive care unit (ICU) remains unclear. Therefore, the present study was conducted to explore this relationship using the Multiparameter Intelligent Monitoring in Intensive Care II (MIMIC-II) database.

## Results

### Analysis of baseline characteristics

The baseline characteristics of the study participants are shown in Table 1. According to the tertiles of 24-hVBGCV, patients were divided into three groups. Statistically significant differences were found among the groups regarding the distribution of age (*P* = 0.005), SOFA score (*P* < 0.001), diabetes (*P* < 0.001), renal failure (*P* < 0.001), and use of insulin (*P* < 0.001). There was no statistical significance regarding sex, ethnicity, obesity, chronic pancreatitis, heart failure, or infection (*P* > 0.05). In the three groups (lowest to highest tertiles), both ICU mortality and in-hospital mortality were significantly different (ICU mortality, 25.3% vs. 32.1% vs. 37.1%, *P* < 0.001; in-hospital mortality, 30.7% vs. 38.0% vs. 42.1%, *P* < 0.001).

**Table 1:** Characteristics of the study subjects.

	24-hVBGCV				*P*
	Tertile 1 (541)	Tertile 2 (542)		Tertile 3 (542)	
Age (years), n (%)					0.005
15–44	84 (15.5)	89 (16.4)		56 (10.3)	
45–59	131 (24.2)	117 (21.6)		108 (19.9)	
60-	326 (60.3)	336 (62.0)		378 (69.7)	
Male, n (%)	292 (54.0)	276 (51.0)		284 (52.5)	0.622
Ethnicity, n (%)					0.118
White	371 (68.6)	369 (68.1)		354 (65.3)	
Black	39 (7.2)	26 (4.8)		46 (8.5)	
Asian	11 (2.0)	15 (2.8)		13 (2.4)	
Hispanic/Latino	6 (1.1)	15 (2.8)		18 (3.3)	
Unknown/other	114 (21.1)	117 (21.6)		111 (20.5)	
Average number of glucose measurements	3.18 ± 1.70	5.49 ± 3.46		7.64 ± 5.68	< 0.001
Mean venous blood glucose level (mg/dl)	134.61	148.04		167.17	< 0.001
Coefficient of variation (%)	6.32	17.51		40.40	< 0.001
Standard deviation	8.62	26.09		69.54	< 0.001
Day 1 SOFA, n (%)					< 0.001
0–9	391 (72.3)	310 (57.2)		247 (45.6)	
10–24	150 (27.7)	232 (42.8)		295 (54.4)	
Obesity	11 (2.0)	9 (1.7)		10 (1.8)	0.901
Diabetes mellitus	94 (17.4)	121 (22.3)		176 (32.5)	< 0.001
Chronic pancreatitis	1 (0.2)	5 (0.9)		2 (0.4)	0.196
Use of insulin	15 (2.8)	24 (4.4)		54 (10.0)	< 0.001
Heart failure	175 (32.3)	194 (35.8)		205 (37.8)	0.163
Renal failure	199 (36.8)	235 (43.4)		274 (50.6)	< 0.001
Infection	67 (12.4)	94 (17.3)		83 (15.3)	0.072
Mortality, n (%)					
ICU mortality	137 (25.3)	174 (32.1)		201 (37.1)	< 0.001
In-hospital mortality	166 (30.7)	206 (38.0)		228 (42.1)	< 0.001

*24-hVBGCV* coefficient of variation of 24-h venous blood glucose, *SOFA* sequential organ failure assessment, *ICU* intensive care unit.

### Relationship between 24-hVBGCV and mortality

In univariate analysis, taking the lowest tertile as a reference, the highest tertile was associated with ICU mortality (OR, 1.738; 95% CI, 1.339–2.256) and in-hospital mortality (OR, 1.640; 95% CI, 1.278–2.106). After adjustment for a series of covariates, the highest tertile was also related to higher ICU mortality (OR, 1.353; 95% CI, 1.018–1.797) and in-hospital mortality (OR, 1.319; 95% CI, 1.003–1.735), compared with the lowest tertile of 24-hVBGCV (Table 2).

**Table 2:** Odds ratios (OR) and 95% confidence intervals (CI) for mortality associated with 24-hVBGCV in overall patients.

	24-hVBGCV			Overall tendency
	Tertile 1 (541)	Tertile 2 (542)	Tertile 3 (542)	
**ICU mortality**				
Events, n (%)	137 (25.3)	174 (32.1)	201 (37.1)	
Unadjusted	1 (Ref)	1.394 (1.070–1.817)	1.738 (1.339–2.256)	1.316 (1.156–1.498)
Adjusted	1 (Ref)	1.278 (0.965–1.694)	1.353 (1.018–1.797)	1.160 (1.007–1.336)
**In-hospital mortality**				
Events, n (%)	166 (30.7)	206 (38.0)	228 (42.1)	
Unadjusted	1 (Ref)	1.385 (1.077–1.782)	1.640 (1.278–2.106)	1.278 (1.129–1.448)
Adjusted	1 (Ref)	1.307 (0.999–1.711)	1.319 (1.003–1.735)	1.146 (1.000–1.313)

Adjusted for age, sex, ethnicity, SOFA score, obesity, infection, diabetes, chronic pancreatitis, heart failure, renal failure, use of insulin. OR was for ICU mortality and hospital mortality.

*24-hVBGCV* coefficient of variation of 24-h venous blood glucose, *ICU* intensive care unit, *SOFA* sequential organ failure assessment.

### Interaction and subgroup analysis

The interaction analyses were conducted between 24-hVBGCV and diabetes. However, there was no significant relationship (*P*_interaction_ = 0.135; hospital, *P*_interaction_ = 0.160), which indicates that whether or not patients have diabetes has no effect on the relationship between 24-hVBGCV and mortality. Considering the clinical practice, the population was also divided into two subgroups: diabetes or non-diabetes patients. Surprisingly, compared with the lowest 24-hVBGCV tertile, the highest tertile was associated with ICU mortality (OR, 1.453; 95% CI, 1.050–2.010) and in-hospital mortality (OR, 1.382; 95% CI, 1.012–1.888) in non-diabetes patients after adjusting for all the covariates (Table 3).

**Table 3:** Odds ratios (OR) and 95% confidence intervals (CI) for mortality associated with 24-hVBGCV in diabetes and non-diabetes groups.

	T1	T2	T3	Overall tendency
**Diabetes**				
Subjective, n (%)	94 (24.0)	121 (30.9)	176 (45.1)	
ICU mortality				
Events, n (%)	24 (19.5)	41 (33.3)	58 (47.2)	
Unadjusted	1 (Ref)	1.495 (0.823–2.716)	1.434 (0.819–2.510)	1.164 (0.890–1.524)
Adjusted	1 (Ref)	1.513 (0.788–2.904)	1.083 (0.583–2.011)	0.994 (0.739–1.337)
**In-hospital mortality**				
Events, n (%)	26 (19.4)	45 (33.6)	63 (47.0)	
Unadjusted	1 (Ref)	1.549 (0.864–2.775)	1.458 (0.844–2.520)	1.171 (0.900–1.523)
Adjusted	1 (Ref)	1.562 (0.821–2.972)	1.082 (0.587–1.993)	0.990 (0.739–1.327)
**Non-diabetes**				
Subjective, n (%)	447 (36.2)	421 (34.1)	366 (29.7)	
ICU mortality				
Events, n (%)	113 (29.0)	133 (34.2)	143 (36.8)	
Unadjusted	1 (Ref)	1.365 (1.015–1.835)	1.895 (1.405–2.557)	1.377 (1.185–1.599)
Adjusted	1 (Ref)	1.224 (0.893–1.677)	1.453 (1.050–2.010)	1.205 (1.025–1.418)
**In-hospital mortality**				
Events, n (%)	140 (30.0)	161 (34.5)	165 (35.4)	
Unadjusted	1 (Ref)	1.358 (1.026–1.797)	1.800 (1.351–2.398)	1.342 (1.163–1.549)
Adjusted	1 (Ref)	1.252 (0.928–1.689)	1.382 (1.012–1.888)	1.176 (1.007–1.375)

Adjusted for age, sex, ethnicity, SOFA score, obesity, infection, diabetes, chronic pancreatitis, heart failure, renal failure, use of insulin. OR was for ICU mortality and hospital mortality.

*24-hVBGCV* coefficient of variation of 24-h venous blood glucose, *ICU* intensive care unit, *SOFA* sequential organ failure assessment.

## Discussion

Many studies have shown that increased MAG change per hour or SD was independently associated with length of ICU stay and mortality in critically ill patients^3,5^. In the present study, we used CV of 24-h venous blood glucose to evaluate the blood GV. Our findings suggest that higher 24-hVBGCV was associated with ICU and in-hospital mortality in patients with acute respiratory failure after adjusting for a series of variables, especially in non-diabetes patients.

Previous studies have focused on the relationship between GV and mortality^2,3,7–9^. By measuring MAG change per hour from 5728 ICU patients in a retrospective cohort^3^, Hermanides J et al. concluded that high MAG variation was strongly associated with increased ICU and in-hospital death; conversely, low GV seemed to have a protective effect, even when mean glucose levels remained elevated. Collecting all glucose values for every patient during the stay in hospital to calculate the mean glucose, Krinsley et al.^5^ found that high GV, calculated by the SD of each patient’s mean absolute glucose level, has an effect on mortality in critically ill adult patients. High SD of glucose may serve as an independent risk factor of ICU mortality^10^. Recently, a prospective observational study^11^, in which GV was calculated as the CV of blood glucose (derived as a percentage of SD to mean blood glucose during the entire ICU stay), also confirmed that patients with lower GV had lower ICU/in-hospital mortality than those with higher GV. However, the population was limited to patients with hyperglycemia and lower SOFA scores; importantly, all blood glucose values from admission to discharge were collected and utilized, which might compromise the feasibility of possible risk stratification in the future.

In the present study, 24-hVBGCV was calculated during the first 24 h after ICU admission, it may provide an earlier prediction of mortality. In the regression model, we adjusted for a series of covariates that may influence mortality; in addition, use of insulin was adjusted into the regression model to eliminate the influence of glucose level. Furthermore, considering the relationship between hyperglycemia and mortality^12^, we conducted a sensitivity analysis to explore whether such a relationship exists in patients with or without diabetes. Surprisingly, compared with the lowest 24-hVBGCV tertile, the highest tertile was associated with ICU mortality and in-hospital mortality in non-diabetic patients after adjustment for all the covariates. Our results are consistent with previously published findings using mean absolute glucose change per hour or SD in critically ill patients^13,14^. Therefore, our findings showed that 24-hVBGCV might serve as an independent risk factor of mortality in patients with acute respiratory failure.

Previous studies have demonstrated that high GV leads to greater oxidative stress and endothelial dysfunction than dose sustained hyperglycemia^15–17^. In vitro, Quagliaro et al.^15^found that exposure of endothelial cells to intermittent high glucose stimulated reactive oxygen species overproduction, leading to accelerated cellular apoptosis. Studies in both patients with diabetes and healthy subjects observed that oscillating glucose produced further significant increases in endothelial dysfunction and oxidative stress compared with sustained high glucose^16,17^. For non-diabetic patients, acute stress response and inflammatory response were the main causes of increased glucose^14^. In addition, El-Osta et al.^18^found that even short-term hyperglycemic spikes could have long-lasting effects on vascular cells. A meta-analysis including at least 12 independent cohorts was shown the relation of glucose variability to ICU mortality in non-diabetic patients with stress hyperglycemia^19^. Diabetic patients may develop a relative tolerance to the cardiovascular effects associated with moderate degrees of hyperglycemia^14^. Moreover, hyperglycemia itself is an independent risk factor for death; thus, death caused by diabetes and its complications might conceal the effect of blood glucose fluctuation on mortality^12^. Therefore, it was reasonable that we found a significant association between GV and mortality in the non-diabetic group rather than in the diabetic group.

This study has the following strengths. First, a large cohort of patients were enrolled from various units (medical and coronary care ICUs), reflecting a real-world situation. Second, this was a homogeneous group of critically ill patients with acute respiratory failure, excluding patients with the extra fluctuations in blood glucose caused by surgical procedures. In addition, the 24-hVBGCV was adopted to avoid the calculation error caused by the difference in blood glucose monitoring frequencies in the treatment process of patients due to diabetes. However, our study also has some limitations. Because of the retrospective design of the study, potential bias (i.e., Berkson’s bias) may exist. Moreover, our study investigated the association between 24-hVBGCV and mortality in patients with acute respiratory failure, rather than causality. Therefore, well-designed prospective studies are needed for future investigation.

## Methods

### Study design

This is a retrospective cohort study using patients’ data from the MIMIC-II database. It was approved by the Institutional Review Boards of Beth Israel Deaconess Medical Center (Boston, MA, USA) and the Massachusetts Institute of Technology (MIT; Cambridge, MA). The patients’ consent was waived because of the retrospective design of the study.

More than 30,000 patients in the MIMIC-II database were enrolled from the intensive care unit (ICU) at Beth Israel Deaconess Medical Center from 2001 to 2008. The database included demographic characteristics, physiological data, treatments, laboratory and radiological data, medical history, and survival outcomes^20^. All data used for this study were de-identified and available upon request. All authors had completed the Collaborative Institutional Training Initiative course at the University of Miami and received permission to access the MIMIC database.

The following measures were extracted for each patient: age at admission, sex, ethnicity, the first 24-h Sequential Organ Failure Assessment (SOFA) score after admission to the ICU, use of insulin, clinical status (obesity, diabetes, chronic pancreatitis, heart failure, renal failure, and infection), survival outcome, and records of IV glucose monitoring during the first 24 h of ICU stay. The extraction of data was performed by SAS version 9.4 (SAS Institute, Cary, NC, USA).

### Participants

All patients (≥ 15 years old) in the database diagnosed with acute respiratory failure coded with International Classification of Diseases, Ninth Revision (ICD-9) were selected for the analysis. Individuals were excluded if (1) their number of 24-h blood glucose measurements was < 2, (2) they had multiple admissions to the hospital or ICU, (3) they stayed in the ICU for less than 24 h, or (4) they had missing covariates.

**Figure 1:**
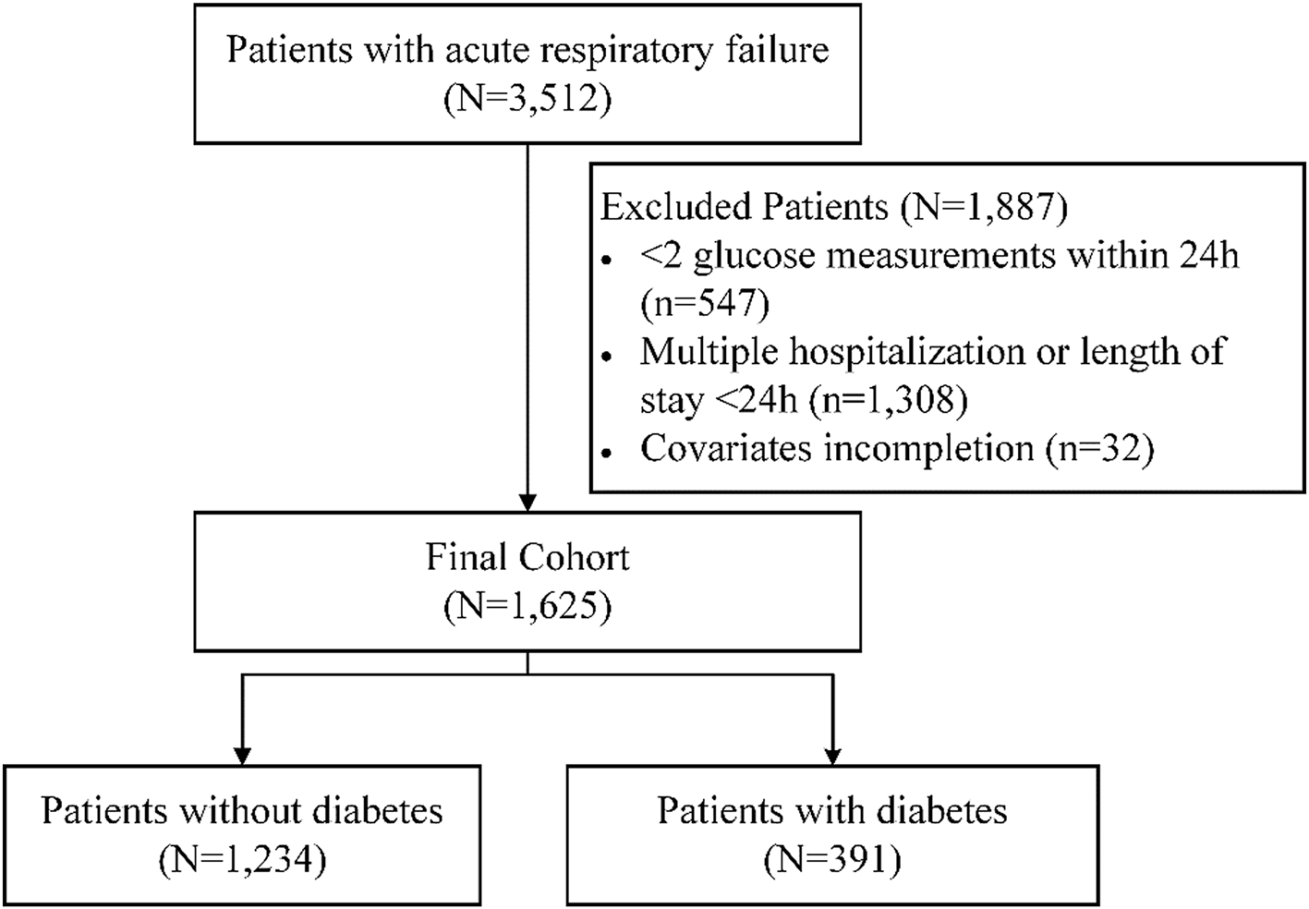
Flow chart of participant selection. A total of 1,625 patients were included in the analysis.

The data of 3512 patients with acute respiratory failure were extracted from the MIMIC-II database. The data of 547 (number of 24-h blood glucose measurements < 2), 1,308 (multiple hospital or ICU admissions and/or length of ICU stay < 24 h), and 32 (missing covariates) patients were excluded. Finally, 1625 patients were included in this study (Fig. 1).

### Evaluation of glycemic variability

We extracted the glucose levels in venous blood samples during the first 24 h of ICU stay. We assessed variation in venous blood glucose using the CV, calculated as the ratio of the SD to the mean venous blood glucose levels, and expressed as a percentage (CV = SD/mean venous blood glucose level × 100%). Patients were divided into three groups according to the tertiles of 24-hVBGCV.

### Outcomes

The outcomes were ICU mortality and in-hospital mortality.

### Statistical analysis

Continuous variables are presented as mean ± SD and compared using analysis of variance (ANOVA). Categorical variables are presented as percentages and compared using a chi-squared test. Logistic regression models were used to assess the impact of 24-hVBGCV on ICU and hospital mortality. In multivariate regression analyses, the adjusted covariates included age, sex, ethnicity, SOFA score, obesity, use of insulin, and related comorbidities that could influence mortality or glucose (heart failure, diabetes mellitus, renal failure, chronic pancreatitis, and infection). Interaction analyses were performed between 24-hVBGCV and diabetes. Subgroup analyses were performed between subjects with and without diabetes. The odds ratios (OR) and their 95% confidence intervals (CI) were generated for logistic regression. All data were analyzed using SPSS version 18.0 (SPSS, Chicago, IL). P values less than 0.05 indicated statistical significance.
